# Return to Sport Activity in the Elderly Patients after Unicompartmental Knee Arthroplasty: A Systematic Review and Meta-Analysis

**DOI:** 10.3390/jcm9061756

**Published:** 2020-06-05

**Authors:** Rocco Papalia, Biagio Zampogna, Guglielmo Torre, Lorenzo Alirio Diaz Balzani, Sebastiano Vasta, Giuseppe Papalia, Antonio De Vincentis, Vincenzo Denaro

**Affiliations:** 1 Deaprtment of Orthopaedic and Trauma Surgery, Campus Bio-Medico University of Rome, Via A. Del Portillo, 21, 00128 Rome, Italy; r.papalia@unicampus.it (R.P.); g.torre@unicampus.it (G.T.); l.diaz@unicampus.it (L.A.D.B.); s.vasta@unicampus.it (S.V.); g.papalia@unicampus.it (G.P.); denaro@unicampus.it (V.D.); 2 Department of Internal Medicine and Gerontology, Campus Bio-Medico University of Rome, Via A. Del Portillo, 21, 00128 Rome, Italy; a.devincentis@unicampus.it

**Keywords:** knee osteoarthritis, unicompartimental knee arthroplasty, sport, activity, elderly

## Abstract

In patients with knee osteoarthritis, when only medial or lateral compartment of the knee is involved, unicompartimental knee arthroplasty (UKA) is a reliable option for addressing the symptoms and restore function. The main aim of the present review is to systematically collect the available evidence concerning the return to sport activity in the elderly patients after UKA. An electronic search was carried out on the following databases; Pubmed-Medline, Cochrane central, and Scopus, searching for randomized controlled trials, prospective cohort studies, retrospective case-control studies, and case series. Data concerning the evaluation of the return to sport (RTS) and of functional outcomes in the elderly patients after UKA surgery. MINORS score was used to assess the risk of methodological biases. Odds ratios and raw proportions were used to report the pooled effect of UKA on the return to sport in comparative and non-comparative studies, respectively. Same level RTS in elderly patients was of 86% (pooled return proportion 0.86, 95%CI 0.78, 0.94), showing also better relative RTS and time to RTS of patients undergoing UKA, in comparison to those undergoing TKA. Sport-specific RTS showed that higher return rates were observed for low-impact sports, whereas high-impact sports prevented a full return to activities. UKA is a valid and reliable option for elderly patients to satisfactorily resume their sport practice, especially for low impact activities. The rate of return to sports following UKA is higher than TKA.

## 1. Introduction

In the present social scenario, the needs of elderly people are changing. It is not infrequent that patients want to stay active and be able to perform physical exercises and sport activities even in an advanced age [1,2]. However, these requests are often undermined by chronic painful conditions, such as osteoarthritis (OA), that do not allow the patient perform all desired activities [3]. In particular, knee OA is a common, debilitating condition that is increasingly widespread accordingly with the aging of the general population [1,2,4,5,6]. It is widely accepted that the definitive treatment for the end stage knee OA is the joint arthroplasty [7]. When only medial or lateral compartment of the knee is involved, unicompartimental knee arthroplasty (UKA) is a reliable option that is raising in popularity [8,9]. Indications to UKA have been widely discussed, but it is well known that this implant provides some advantages: lower invasiveness, shorter rehabilitation time, restoration of a wider range of motion, and physiological proprioception of the knee due to cruciate ligaments retention [10]. Several studies in literature reported the benefits of patients who underwent UKA in terms of pain relief and quality of life, with a good to excellent return to activities [11]. The opportunity to move and walk without pain, with also a good recovery of the motion, allows individuals to perform physical activity and sport, which is particularly important to prevent systemic diseases associated to sedentary life such as obesity, diabetes, cardiovascular accidents, and cancer [12,13,14]. One of the principal expectation for active patients before undergoing UKA surgery, is about their chances to perform physical activity and sport after surgery. Moreover, active patient is mostly interested in type and level of sport activity [15,16]. The current scientific literature answers those questions mainly with recommendations based on expert opinions and surgical society guidelines [17,18], but still lacks high level evidence-based guidelines, especially regarding the elderly population. The main aim of the present manuscript is therefore to systematically collect the available evidence concerning the return to sport activity in the elderly patients after UKA, with a special concern to the type of activity. A secondary endpoint of this investigation is to assess the functional outcomes in the same population.

## 2. Methods

A systematic review and meta-analysis was carried out using the Preferred Reporting Items for Systematic Reviews and Meta-analysis (PRISMA) guidelines [19]. The review was planned and conducted following the PRISMA checklist. According to PICO, the following elements have been used to frame the study question.

Population: Elderly patients.

Intervention: Unicompartmental Knee Arthroplasty (UKA).

Comparison: Total Knee Arthroplasty (TKA) or no comparison.

Outcomes: Return to sport activity and functional outcomes.

### 2.1. Criteria for Considering Studies for This Review

The studies considered for inclusion were randomized controlled trials (CRT), prospective cohort studies (PCS), retrospective case–control studies (RCS), and case series (CS). The main topic of the papers had to be the evaluation of the return to sport activity and of functional outcomes in the elderly patients after UKA surgery. Case Reports, Reviews, and Meta-analyses were not eligible for inclusion. Moreover, in vitro studies and cadaver studies were excluded from the review analysis. Given the specific focus on a selected population, only studies reporting outcomes of patients aged 65 or older were considered for inclusion (average population age > 65). 

### 2.2. Primary Outcome Measures

The absolute numbers and proportions of patients returned to same level sport activities (RTS) was considered as the primary outcome measure and assessed throughout the included studies. Sport-specific return was extracted from the studies, to stratify results according to the type of activities.

### 2.3. Secondary Outcome Measures

The secondary endpoint was achieved by evaluating the following measures; Oxford Knee Score (OKS), Knee injury and Osteoarthritis Outcome Score (KOOS), Knee Society Score (KSS) and American Knee Society Score (AKSS), University of California Los Angeles (UCLA), Tegner and Lysholm scales, and the Western Ontario McMaster universities osteoarthritis index (WOMAC). These measures were evaluated across the included studies. 

### 2.4. Search Strategy for Study Identification

**Figure 1:**
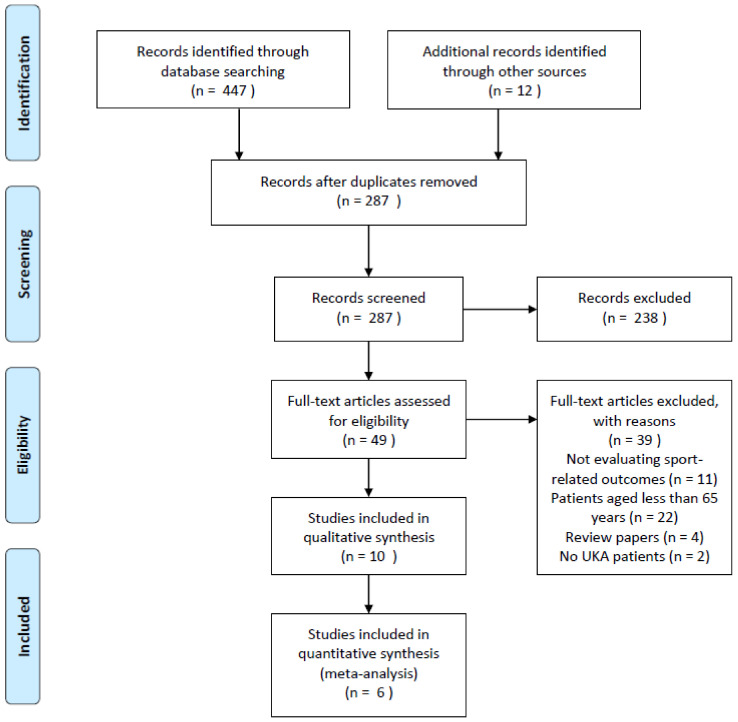
Study selection flowchart (UKA: Unicompartimental Knee Arthroplasty).

The online search was carried out on the following databases; Pubmed-Medline, Cochrane central, and Scopus. The following search string was used; (Arthroplasty, Replacement [MeSH Terms]) AND joint, knee [MeSH Terms]) AND sports [MeSH Terms] and (arthroplasties, knee replacement [MeSH Terms]) AND sports [MeSH Terms]. The bibliography of the included studies and of recent review articles was screened for further relevant articles, potentially missed at the electronic search. After duplicates removal, all the retrieved studies were firstly screened by title to find studies dealing with UKA. Two independent reviewers (B.Z. and G.T.) evaluated the abstract of each of the papers considered for inclusion. Discordant opinions concerning study inclusion were discussed with a third experienced reviewer (R.P.). If abstract was not sufficient to define inclusion of a paper, the full-text was retrieved and evaluated. Articles included for review process were retrieved in full-text and read. The search process was summarized in Figure 1.

### 2.5. Data Collection and Analysis

Data were extracted independently by two reviewers (B.Z. and L.A.D.B.) and tabulated according to primary and secondary outcomes of this review. Discordant opinions in data extraction were solved by discussion with a third reviewer (R.P.).

### 2.6. Risk of Bias Assessment

The quality of the included non-randomized studies was independently evaluated by two reviewers (L.D.B. and B.Z.) using the Methodological Index for Non-randomized Studies (MINORS) score. [20] The following domains were assessed; a clearly stated purpose, inclusion of consecutive subjects, prospective data collection, endpoints appropriate to the purpose of the study, unbiased assessment of the study endpoints, follow-up period appropriate for the study, loss to follow-up of less than 5%, prospective calculation of the study size, adequate control group, contemporary group, baseline group equivalence, and adequate statistical analysis. The last four items are specific to comparative studies. Each item was scored from 0 to 2 points, with a global ideal score of 16 points for non-comparative studies and 24 points for comparative studies. 

### 2.7. Quantitative Analysis

Meta-analysis was carried out to investigate the effect of UKA on the return to sport activity either in comparison with TKA or in non-comparative studies. Furthermore, return to specific sport activities was pooled if at least three studies reported the same sport. Odds ratios (ORs) and raw, i.e. untransformed, proportions were used to report the pooled effect of UKA on the return to sport probabilities in comparative (vs. TKA) and non-comparative studies, respectively. Heterogeneity was evaluated using Q statistic, expressed as the p value for the χ test under the null hypothesis that the between-study variance (τ) equals 0, and I2 test. All the conducted meta-analyses evidenced the presence of significant heterogeneity, defined as a I2 > 55% and/or a Q statistic p value below 0.05. Accordingly, random effect models were applied. Finally, the likelihood of methodological bias among included studies was estimated with the visual inspection of the funnel plot. Analyses were conducted using metafor and meta packages in R 3.6.1 (R Foundation for Statistical Computing, Vienna, Austria). 

## 3. Results

### 3.1. Research Results

Electronic search identified 447 papers, and of these 287 scientific products were screened for analysis. Full text of 49 papers was accurately analyzed and 28 were excluded for following reasons; absence of postoperative sport-related outcomes, cohort mean age lower than 65 years old, duplicated papers and no UKA patients. Finally, 10 [10,21,22,23,24,25,26,27,28,29] articles were included according PRISMA selection process (Figure 1).

### 3.2. Included and Excluded Studies

Among 10 studies, only 2 study were prospective [24,26], 6 evaluated the cohort retrospectively (LOE III) [21,22,23,27,28,29], and 2 were case series (LOE IV) [10,25]. Five studies out of 10 compared clinical outcome of UKA and TKA cohorts [22,23,24,28,29]. Six studies reported specific RTS outcome like preoperative and postoperative sport participation, RTS rate, time to RTS and pre and postoperative sport-specific participation [10,21,22,27,28,29]. Canetti et al. compared two different cohort of lateral UKA performed with and without robotic assistance [21].

### 3.3. Demographic Results

Overall number of patients analyzed in the present review was 5220 with 2930 UKA and 2447 TKA implanted. Mean age of UKA’s was 66.3, whereas mean age of TKA cohort was 74 years old. Mean follow-up was 2.1 years. In three studies type of prosthesis was not specified [22,24,29] while 4 papers reported outcome of mobile bearing UKA [25,26,27,28]. Demographic parameters of included study are summarized in Table 1.

### 3.4. Return to Sport Activity

**Figure 2:**
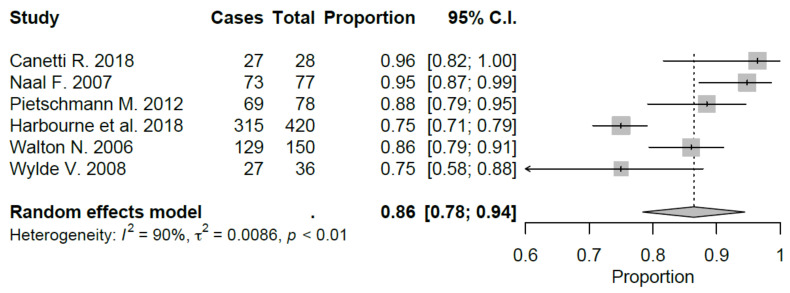
Overall return to sport after UKA (C.I.: Confidence Intervals).

**Figure 3:**
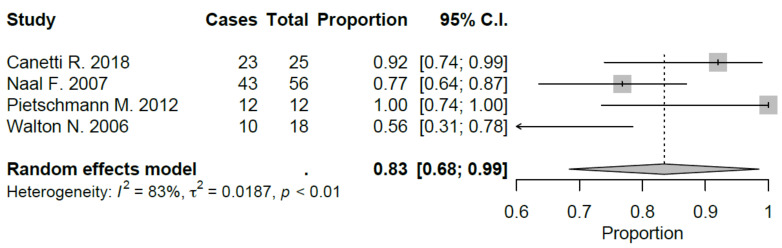
Return to hiking after UKA (C.I.: Confidence Intervals).

**Figure 4:**
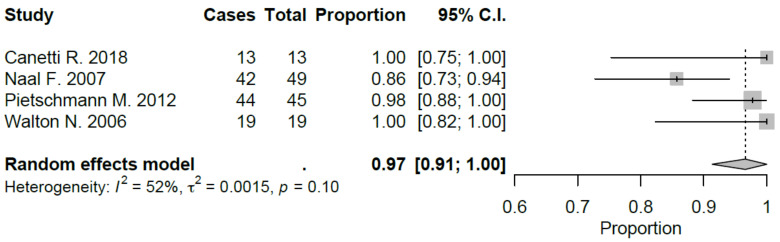
Return to cycling after UKA (C.I.: Confidence Intervals).

**Figure 5:**
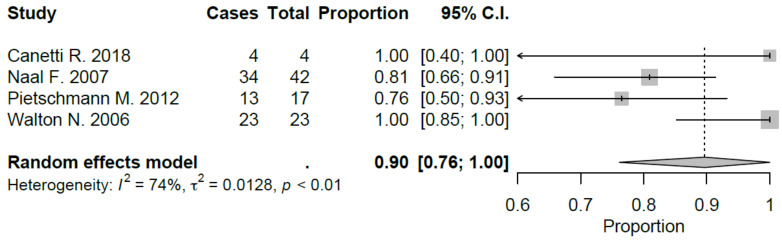
Return to swimming after UKA (C.I.: Confidence Intervals).

**Figure 6:**
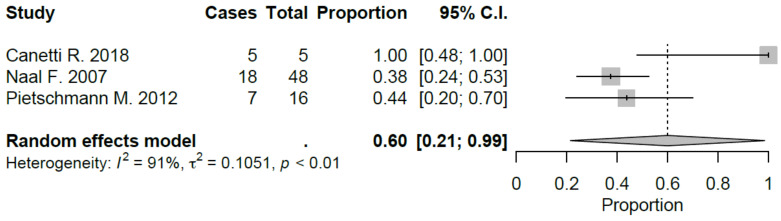
Return to alpine ski after UKA (C.I.: Confidence Intervals).

**Figure 7:**
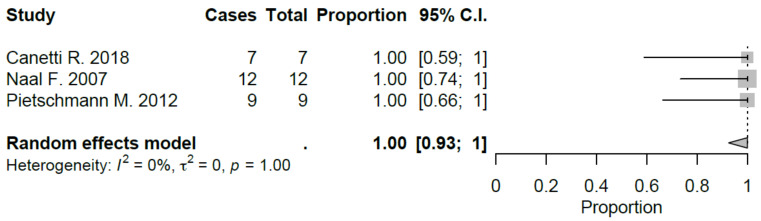
Return to fitness after UKA (C.I.: Confidence Intervals).

**Figure 8:**
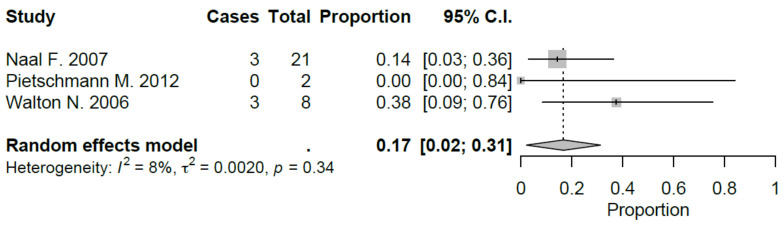
Return to tennis after UKA (C.I.: Confidence Intervals).

Sport-specific return rates were analyzed in 50% of the studies included [10,21,22,27,28,29]. Mean RTS rate for UKA was 89.5%, ranging from 75% [22,29] to 100% [28]. Mean preoperative sport participation rate was 71.8% of the patients, ranging from 36% [29] to 100% [21], and mean postoperative sport participation rate was 70.2% of the patients, ranging from 27% [29] to 100% [21]. Mean time to RTS was 6.2 months. Results of the study published by Canetti et al. [21], showed for UKA robotic assisted group a statistically significant difference in terms of time to return to sport compared to conventional UKA with a similar RTS rate (100% vs. 94%). The cohort of medial UKA of Pietschmann et al. [27] had an 88% of RTS rate with 80.1% of patients that returned to preoperative activity level. Naal et al. [10] reported RTS rate of 95%; moreover, the activity frequency (session per week) was maintained in postoperative assessment (2.9 vs. 2.8) with a slight decrease in terms of session length (66 vs. 55 minutes). Overall, by meta-analyzing available studies, we evidenced a good proportional RTS (0.86 95% CI 0.78, 0.94), with sport-specific RTS favoring those sports with low-impact. Meta-analysis results were showed in Figure 2, Figure 3, Figure 4, Figure 5, Figure 6, Figure 7 and Figure 8.

### 3.5. Comparison with TKA

**Figure 9:**
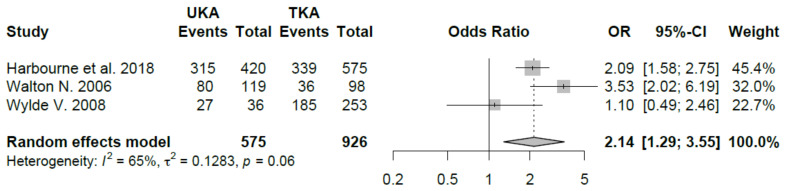
Return to sport after UKA vs. TKA (C.I.: Confidence Intervals, O.R.: Odds Ratio)**.**

Three papers compared RTS outcome of UKA and TKA patients’ cohort [22,28,29]. Harbourne et al. [22] at 12 months of follow-up recorded a higher rate of return to activity in patients with UKA than TKA (75% vs. 59% *p* < 0.001). According to results of Walton et al. [28], the UKA group had a better percentage of patients that increased or maintained sport activity compared to the TKA group (P_.0003); moreover, TKA patients significantly reduced postoperative sport activity compared to UKA’s (P_.0001). Wylde et al. [29] investigated return to sport after different type of implant (THA, Hip Resurfacing, TKA, UKA) and no significant difference differences were detected in postoperative sport participation between UKA and TKA (75% vs. 73.1%). Meta-analysis study pooling showed a better RTS in patients undergoing UKA (Odds Ratio 2.14 95% CI 1.29, 3.55). Results are shown in Figure 9.

### 3.6. Clinical Outcome Data

Several clinical outcome assessment measure and sport specific questionnaires were utilized. Oxford Knee score were used in six studies [22,24,25,26,27,28], Knee Society Score (KSS) in five studies [10,21,24,25,26,27], WOMAC in three studies [21,24,27,29], Forgotten Joint Score (FJS) and UCLA in two studies [21,24], Tegner Activity Score in two studies [25,26], KOOS [23] and Lysholm Knee Scale [21] in one study. Clinical results of Canetti et al. [21] showed a higher IKSS-Objective (97.2 ± 5.9 vs. 91.2 ± 6.5; *p* < 0.05) and a higher IKSS-Objective improvement (+ 30.9 ± 7.7 vs. + 22.8 ± 12.2; *p* < 0.05) compared to conventional group of lateral UKA. Naal et al. [10] obtained an improvement on KSS score in their postoperative assessment (from 129.9 ± 24.8, vs. to 186.9 ± 18.3) with a good result in terms of quality of life stated with SF-36. Pandit et al. [26] obtained a good postoperative results with the first 1000 cases of Oxford phase 3 medial UKA at 5 years: mean OKS was 41.3 (SD 7.2), mean AKS Objective Score 86.4 (SD 13.4), mean AKS Functional Score 86.1 (SD 16.6), and mean Tegner activity score 2.8 (SD 1.1). The same group, in 2015 [25], published results of cementless fixation for the same implant with similar postoperative clinical and functional results: OKS 43 (SD 7), AKSS (objective) 81 (SD 13), AKSS (functional) 86 (SD 17), and Tegner activity score of 3 (1–8). Pietschmann et al. [27] had a higher postoperative OKS, KSS, WOMAC and UCLA score. Active patients in sport preoperatively, except for KSS knee objective score, obtained statistically significant higher score than inactive patients group (OKS < 0.01, UCLA < 0.0001, KSS function < 0.01, KSS knee subjective < 0.01, KSS overall < 0.01, WOMAC < 0.05, WOMAC stiffness < 0.05, WOMAC ADL < 0.01, WOMAC overall < 0.01). Results are summarized in Table 2. 

### 3.7. Clinical Outcome Data UKA vs. TKA

Four studies compared clinical outcome in patients aged more than 65 years old and underwent to UKA and TKA [23,24,28,29]. In the patients’ cohort of Lygre et al. [23], UKA had a statistically significant superiority over TKA in terms of KOOS “Symptoms” (adjust mean diff 2.7 *p* = 0.04), KOOS “Function in Daily Living” (ADL) (adjust mean diff 4.1 *p* = 0.01) and KOOS "Function in Sport and Recreation" (adjust mean diff 5.4 *p* = 0.006). A prospective study designed by Matthews et al. [24] showed no statistical difference according to satisfaction (89 vs. 87 *p* = 0.41) and perception of knee normality (69 vs. 68 *p* = 0.99) scores between UKA and TKA; nevertheless, UKA reached a statistically significant higher WOMAC (*p* = 0.003), SF-36 (physical *p* < 0.001; mental *p* = 0.25), Oxford knee (*p* < 0.001), American Knee Society (clinical *p* = 0.002; function *p* < 0.001) and Total Knee Function Questionnaire scores (ADL *p* = 0.002; sport and exercise *p* = 0.02; movement and lifestyle *p* = 0.02). Walton et al. [28] compared in their study Mini-Incision Unicompartmental Knee Arthroplasty versus TKA and reported better results in terms of OKS (*p* = 0.0426) and mean modified Grimby score (3.89 SD:1.27 vs. 2.76 (SD:1.12). In the last comparative study, performed by Wylde et al. [29], no clinical difference between UKA and TKA in terms of WOMAC pain (81.5 SD = 20.8 vs. 81.6 SD = 19.3) and function (76.3 SD = 21.4 vs. 79.1 SD = 20.5).

### 3.8. Quality Assessment (MINORS)

**Figure 10:**
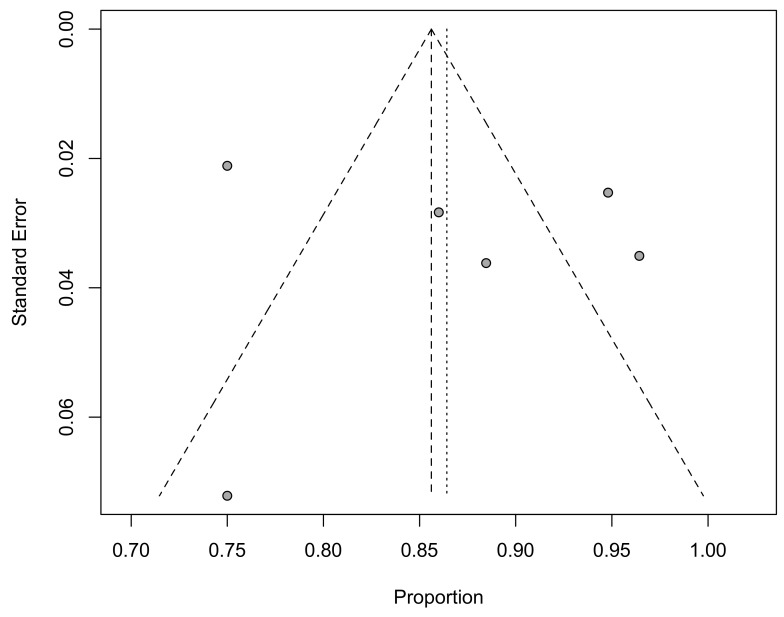
Funnel plot showing studies evaluating RTS after UKA.

The MINORS score ranged from 7 [27] to 12 [25] for non-comparative studies and from 12 [28] to 17 [21] for the comparative ones (Table 1). The mean value was 10 for non-comparative studies and 15 for comparative studies. The funnel plot of studies evaluating RTS after UKA showed a symmetrical distribution, while a rather poor precision of observations, suggesting an overall low-moderate risk of methodological bias (Figure 10).

## 4. Discussion

Elderly patients represent a selected population, which is changing in activity needs in recent years, according to general lifestyle modifications of the society. The main findings of the present investigation suggest a good proportional same level RTS in elderly patients after UKA (return proportion 0.86, 95%CI 0.78, 0.94), showing also better relative RTS and time to RTS of patients undergoing UKA, in comparison to those undergoing TKA. Moreover, patients undergoing TKA were more likely to reduce their activity level after the surgery [28]. Meta-analysis of the sport-specific RTS showed that higher return rates were observed for low-impact sports (e.g., swimming, fitness, hiking), whereas high-impact sports (e.g., tennis and alpine ski) prevented a full return to activities. The proportion of RTS for cohorts of patients undergoing UKA is in line with authors’ experience and with literature-reported rates [10,21,30]. However, given the high heterogeneity of the studies concerning this outcome (90%), the result should be carefully interpreted. A first consideration concerns the average age of the cohorts, which was higher in those study reporting a lower RTS proportion. Furthermore, the differences in type of UKA implants could determine the activity level, with possible implications in polyethylene wearing [31]. Another major concern in general study heterogeneity is the absence, in almost all the studies, of a description of rehabilitation protocols and of surgical incision. In an era of wide differences in rehabilitation (i.e., fast-track, aquatic rehabilitation), understanding the post-operative protocols may be the key to evaluate the postoperative outcomes [32]. Moreover, the knee extension apparatus, thus the surgical approach plays a major role in return to activity and its timing.

Clinical outcomes reported in the included studies were filtered to collect those relative to sport participation and activity level. Although heterogeneous, an overall evaluation of the scores showed that either objective and subjective assessment improved significantly after UKA, suggesting that patient perception of the beneficial effects of the surgery reflects a standardized clinical examination and ROM assessment. Similarly, comparison of clinical outcomes after UKA with those after TKR favored the patients undergoing UKA. However, given the absence of control groups in most of the included studies, the meta-analysis evaluation was not possible, given the impossibility to calculate the standardized mean difference.

Concerning comparison of RTS and clinical outcomes between cohorts undergoing UKA and TKA, a potential confounding factor is age, as average age of UKA cohorts was 66.3 years, while mean age of TKA cohorts was 74 years. A 10-year difference is relevant by the observation that younger patients are more likely to continue in sport participation. This is especially true, given the higher and higher percentage of elderly population involved in sport activities in recent years [33]. Focusing on elderly population underlined some differences with available evidence on general population (non-elderly) [34,35]. First of all, as reported in previous literature reviews [11], the return to high-impact sport in patients that underwent either TKA and UKA was high, and not excessively different from those of low-impact sports [36,37]. Common experience leads the orthopedic surgeon to suggest caution in sport participation after joint arthroplasty, for the risk of component migration, loosening, and periprosthetic fractures. However, no specific evidence advices about long term results of sport involvement after UKA [30].

The UKA, a joint preserving arthroplasty, has been designed for those patients with localized osteoarthritis and was found especially beneficial in active individuals. However, the follow-up length reported in available literature is too short to assess failure and revision rates. It is opinion of the authors that until clear data will be available, the general attitude for patients and surgeon in regard to RTS will go toward a careful approach.

Another concern, in regards of the available literature, is the lack of information about reasons of not to return to sport activities. None of the articles included in this review reported the clinical and functional reasons that prevented the patients to return to activity, except a few reported surgical complications (i.e., common peroneal paralysis [21]). The issue of motivational causes that prevent RTS is relevant and only some studies in the gerontology field address the question [6,14]. An interesting point to improve future research would be to introduce motivational and depression scales next to functional patient reported outcome measures.

The overall LOE of included studies was low, as most of the evaluations were retrospective or were case series. This is reflected in the relatively high risk of bias which MINORS score showed. In particular, the worst item was the blinding of participants. However, given the type of therapeutic intervention considered, blinding was impossible.

The funnel plot evaluation was limited to those studies assessing the proportional RTS of patients after UKA. A balanced funnel was observed at visual inspection, but a relatively low precision was found for some studies, suggesting an overall low–moderate risk of methodological bias.

This is the first literature review and meta-analysis that selected age of the cohorts undergoing UKA, focusing the research questions on the elderly population. Meta-analysis had a two-fold aim: to investigate proportional RTS in UKA-only cohorts and to compare UKA and TKA patients. However, the study was not free from limitations. First of all, the limited number of studies reporting sport-specific outcomes after UKA and the low LOE prevented the authors to gather a sufficient evidence to finally answer to the research questions. Furthermore, the differences in study design, age of the cohorts and effect sizes yielded to high heterogeneity and prevented to draw a robust overall meta-analysis.

## 5. Conclusions

UKA is a valid and reliable option for elderly patients to satisfactorily resume their sport practice, especially for low impact activities. The rate of return to sports following UKA is higher than TKA. The most practiced sports after surgery are low contact activities such as swimming, fitness, and hiking. Unfortunately, there is a lack of consistent clinical data on the functional improvement before and after surgery in elderly patients, thus a standardized evaluation of patient after surgery is prevented. More, prospective, comparative studies are needed to determine the standardized functional improvement of elderly patients after UKA.

## Figures and Tables

**Table 1:** Demographic studies details.

Author and Year	LOE	N.er of Patients	M	F	Type of Arthroplasty	Type of Implant	Bearing and Fixation	Mean Age at Surgery	Mean Follow-up (Months)	MINORS Score
Canetti et al. 2018	3	25 (28 knees)	9	21	Robotic UKA 11	Resurfacing Uni Evolution, Tornier; BlueBelt Navio robotic surgical system.	Fixed bearing and cemented.	66.5 ± 6.8	34.4 ± 10.5	17
					______			___	___	
					Conventional UKA 17			59.5 ± 9.9	39.3 ± 15.5	
Harbourne et al. 2018	3	995	44	54	UKA 420	n/a	n/a	67 ± 10	12	16
			__	__	_____			___		
			6	9	TKA 575			70 ± 9		
Lygre et al. 2010	3	1344	152	220 690	UKA: 372	Genesis Uni (Smith & Nephew), Miller-Galante all poly Uni (Zimmer), Oxford III (Biomet).	Fixed/mobile and cemented.	68.8 ± 8.8	58.8 ± 27.6	15
			_		____	____	___	___	___	
			282		TKA: 972	AGC (Biomet), Genesis I (Smith & Nephew), LCS (DePuy) and NexGen (Zimmer).	Fixed bearing and cemented.	76 ± 7.7	84 ± 28.8	
Matthews et al. 2013	2	68	10	24	UKA 34	n/a	n/a	UKA 67.3 ± 9.1	12	15
			_	_	____			___		
			14	20	TKA 34			TKA 69.2 ± 7.7		
Naal et al. 2007	4	83 (83)	45	38	UKA	Preservation prosthesis (DePuy).	Fixed bearing and cemented.	65.5 ± 9.1 years (47–83)	12	10
Pandit et al. 2011	4	818 (1000)	-	-	UKA	Phase 3 Oxford medial UKA (Biomet).	Mobile bearing and cemented.	66 (32–88)	67.2	11
Pandit et al. 2015	4	579 (520)	299	221	UKA	Phase 3 Oxford medial UKA (Biomet).	Mobile bearing and cementless	65.1 ± 10.3 (35–94)	40.8 ± 20.4	12
Pietschmann et al. 2012	3	131	57	74	UKA	Phase 3 Oxford medial UKA (Biomet).	Mobile bearing and cemented.	65.3 (44–90)	50.4 (12–120)	7
Walton et al. 2010	3	150 (183)	76	74	UKA	Phase 3 Oxford medial UKA (Biomet).	Mobile bearing and cemented.	71.5 ± 9.85	12	12
		_	_	_	_	_	_	_		
		120 (142)	61	59	TKA	n/a	n/a	71.53 ± 9.87		
Wylde et al. 2008	3	966	48 511	52 355	UKA: 100 TKA: 866	n/a	n/a	66 (45–88) 69.6 (26–93)	24	16

UKA: Unicompartmental Knee Arthroplasty TKA: Total Knee Arthroplasty (TKA), LOE: Level: of Evidence, M: Males, F: Females.

**Table 2:** Functional outcomes of included studies.

Author and Year	N.er of Patients	Preoperative		Postoperative		Outcome
		Mean	SD	Mean	SD	
Canetti R. 2018	28	66.3	8.9	97.2	5.9	KSS-Objective
		84.6	11.3	96.4	9.2	KSS-Functional
		6.4	1.6	6.6	1.4	UCLA
		n/a	n/a	96.4	8.3	Lysholm scale
Naal F. 2007	83	129.9	24.8	186.9	18.3	KSS total
Pandit H. 2011	1000	24.7	8.7	38.6	8.4	OKS
		2.3	1.1	2	8.4	Tegner activity score
		68.7	18	81.1	11.3	AKSS-F
		47.4	20	81.1	16	AKSS-O
Pandit H. 2015	520	27	9	43	7	OKS
		3	n/a	3	n/a	Tegner activity score
		71	17	86	16	AKSS-F
		52	20	92	12	AKSS-O
Pietschmann M. 2012	131	n/a	n/a	95.3	9.5	KSS-Objective
		n/a	n/a	86.7	13.8	KSS-Functional
		n/a	n/a	6	1	UCLA
		n/a	n/a	189	26.8	KSS total
		n/a	n/a	38.6	7.3	OKS
		n/a	n/a	90.6	9.7	WOMAC ADL

S.D.: Standard Deviation, N/A: Not Available, OKS: Oxford Knee Score, KOOS: Knee injury and Osteoarthritis Outcome Score, KSS: Knee Society Score, AKSS: American Knee Society Score, UCLA: University of California Los Angeles, WOMAC: Western Ontario McMaster universities osteoarthritis index.
